# Relation of Serum Serotonin Levels to Bone Density and Structural Parameters in Women

**DOI:** 10.1359/jbmr.090721

**Published:** 2009-07-13

**Authors:** Ulrike I Mödder, Sara J Achenbach, Shreyasee Amin, B Lawrence Riggs, L Joseph Melton, Sundeep Khosla

**Affiliations:** 1 Endocrine Research Unit, College of Medicine, Mayo Clinic Rochester, MN, USA; 2 Department of Health Sciences Research, College of Medicine, Mayo Clinic Rochester, MN, USA; 3 Division of Rheumatology, College of Medicine, Mayo Clinic Rochester, MN, USA

**Keywords:** serotonin, bone density, bone structure, SSRI, osteoporosis

## Abstract

Recent studies have demonstrated an important role for circulating serotonin in regulating bone mass in rodents. In addition, patients treated with selective serotonin reuptake inhibitors (SSRIs) have reduced areal bone mineral density (aBMD). However, the potential physiologic role of serotonin in regulating bone mass in humans remains unclear. Thus we measured serum serotonin levels in a population-based sample of 275 women and related these to total-body and spine aBMD assessed by dual-energy X-ray absorptiometry, femur neck total and trabecular volumetric BMD (vBMD) and vertebral trabecular vBMD assessed by quantitative computed tomography (QCT), and bone microstructural parameters at the distal radius assessed by high-resolution peripheral QCT (HRpQCT). Serotonin levels were inversely associated with body and spine aBMD (age-adjusted *R* = −0.17 and −0.16, *P* < .01, respectively) and with femur neck total and trabecular vBMD (age-adjusted *R* = −0.17 and −0.25, *P* < .01 and < .001, respectively) but not lumbar spine vBMD. Bone volume/tissue volume, trabecular number, and trabecular thickness at the radius were inversely associated with serotonin levels (age-adjusted *R* = −0.16, −0.16, and −0.14, *P* < .05, respectively). Serotonin levels also were inversely associated with body mass index (BMI; age-adjusted *R* = −0.23, *P* < .001). Multivariable models showed that serotonin levels remained significant negative predictors of femur neck total and trabecular vBMD, as well as trabecular thickness at the radius, after adjusting for age and BMI. Collectively, our data provide support for a physiologic role for circulating serotonin in regulating bone mass in humans. © 2010 American Society for Bone and Mineral Research

## Introduction

Recent elegant genetic studies in rodents have demonstrated a key role for circulating serotonin (5-hydroxytryptamine, 5-HT) in regulating bone formation and skeletal mass.(1) These studies have shown that low-density lipoprotein (LDL)-receptor-related protein 5 (LRP5), which is assumed to be a coreceptor for WNT proteins,(2) may regulate bone formation and bone mass not through direct effects on osteoblasts but rather indirectly by suppressing serotonin production in the duodenum via inhibition of the expression of tryptophan hydroxylase 1 (Tph1), the rate-limiting enzyme for serotonin synthesis.(1) Serotonin appears to act on osteoblasts through the 5-hydroxytryptamine receptor 1b (Htr1b) and inhibits their proliferation. Consistent with this, circulating serotonin levels that were decreased by means of a low-tryptophan diet normalized bone formation and bone mass in LRP5-deficient mice, and gut- but not osteoblast-specific LRP5 inactivation decreased bone formation in a β-catenin-independent manner. Conversely, gut-specific activation of LRP5 or inactivation of Tph1 increased bone mass and prevented ovariectomy-induced bone loss.(1)

These findings in rodents are consistent with the limited data in humans that suggest an important role for serotonin in regulating bone metabolism. Thus Yadav and colleagues(1) extended their findings in rodents to humans with LRP5 loss-of-function mutations, which cause the osteoporosis pseudoglioma (OPPG) syndrome characterized by severely reduced bone mass, and showed that serum serotonin levels were elevated at approximately 250 ng/mL in the sera of the three OPPG patients analyzed as compared with control subjects, who had a mean serum serotonin level of approximately 50 ng/mL. Conversely, platelet-poor plasma serotonin levels were suppressed in two subjects with high bone mass owing to activating mutations in LRP5.(1)

Further support for an important role for serotonin in bone metabolism comes from studies in patients treated with selective serotonin reuptake inhibitors (SSRIs), which increase extracellular serotonin levels. Thus, in data from the Study of Osteoporotic Fractures (SOF), use of SSRIs, but not tricyclic antidepressants, was associated with increased rates of bone loss at the hip.(3) Similarly, use of SSRIs, but not other antidepressants, was associated with reduced BMD at multiple skeletal sites in men.(4) Finally, recent data indicate that daily SSRI use was associated with a twofold increase in the risk of clinical fragility fractures after adjustment for potential covariates.(5) Consistent with these findings, both deletion(6,7) and inhibition(7) of the serotonin (5-HT) transporter in rodents is associated with reduced bone mass.

Despite these findings in rodents and in patients treated with SSRIs, there are currently no data in subjects not being treated with these agents regarding a possible physiologic role for circulating serotonin levels in regulating bone turnover or mass in humans. Thus we measured serum serotonin levels in a population-based cohort of women and related these to anthropometric measures, bone turnover markers, and bone density/structural parameters at multiple skeletal sites using a combination of dual-energy X-ray absorptiometry (DXA), quantitative computed tomography (QCT), and high-resolution peripheral QCT (HRpQCT).

## Methods

### Study subjects

We recruited 375 women from an age-stratified random sample of Rochester, Minnesota residents, who were selected using the medical records linkage of the Rochester Epidemiology Project.(8) This population is highly characteristic of the white population of the United States, but blacks, Asians, and Hispanics are underrepresented. The sample spanned ages from 21 to 97 years. For the present analysis, we excluded 99 women on medications that potentially could affect circulating serotonin levels (i.e., SSRIs, adrenergic blockers, adrenergic stimulants, alpha blockers, alpha-adrenergic agonists, anticholinergic agents, anticonvulsants, antidepressants, antipsychotics, beta blockers, and gastrointestinal prokinetic agents) or on corticosteroids, bisphosphonates, and selective estrogen receptor modulators. One woman with a very high serotonin level [402 ng/mL, >4 standard deviations (SDs) above the mean] also was excluded. The analysis thus was based on 275 women: 90 of these were premenopausal, 125 were postmenopausal and were not taking hormone therapy (HT, defined as oral or transdermal estrogen preparations with or without a progestin), and 60 were postmenopausal and taking some form of HT. We defined menopause as the absence of menses for greater than 6 months. Reflecting the ethnic composition of the community, 98.5% of the women were white. All studies were approved by the Mayo Institutional Review Board, and written informed consent was obtained from all subjects prior to evaluation.

### Study protocol

Subjects were admitted to the outpatient Clinical Research Unit following an overnight fast. They consumed their habitual diet the day prior to study without any dietary restrictions. Height (m) and weight (kg) were measured using a customized height gauge (Mayo Section of Engineering) and an electronic weight scale (Model 5002, Scale-Tronix, Inc., White Plains, NY, USA), and body mass index (BMI) was defined as killiograms per square meter (kg/m^2^). Following a blood draw, the subjects underwent the various imaging procedures described below.

### Sample collection

The samples for this study were collected from December 2000 through March 2004. Venous blood was withdrawn into uncoated clot tubes (BD Biosciences, San Jose, CA, USA). After agglutination, the blood was centrifuged at room temperature for 10 minutes at 1000 × g. The serum was divided into 0.5 mL aliquots and immediately frozen at −80°C until used for different assays. The samples were thawed immediately before use, and only previously unthawed aliquots were used for the serotonin assay. A previous study from our group(9) has demonstrated the stability of osteocalcin, a relatively unstable protein,(10) under our collection and storage conditions.

### Serum measurements

Serum serotonin levels were measured using a competitive enzyme-linked immunosorbent assay (ELISA; Immuno-Biological Laboratories, Inc., Minneapolis, MN, USA; interassay coefficient of variation 6%). Serum calcium and phosphorus levels were measured by autoanalyzer (Roche Diagnostic Corp., Indianapolis, IN, USA; interassay coefficient of variation 5%). Serum 25-hydroxyvitamin D [25(OH)D] was measured by a competitive protein-binding assay (Nichols Institute Diagnostics, Capistrano, CA, USA; interassay coefficient of variation <15%), and serum parathyroid hormone (PTH) was measured using a two-site immunoassay for intact PTH (Diagnostic Products Corporation, Los Angeles, CA, USA; interassay coefficient of variation <13%). Serum osteocalcin was measured using a two-site immunoradiometric assay (CIS-US, Bedford, MA, USA; interassay coefficient of variation 8%). Serum amino-terminal propeptide of type I collagen (PINP) was measured by radioimmunoassay (DiaSorin, Stillwater, MN, USA; interassay coefficient of variation <9%). Serum cross-linked C-telopeptide of type I collagen (CTx) was measured using an ELISA (Nordic Biosciences, Herlev, Denmark; interassay coefficient of variation <10%).

### Total-body DXA

This study was designed originally to focus on volumetric QCT parameters and did not include site-specific DXA measures of the spine or hip. However, the subjects did have a total-body DXA performed (Prodigy, GE Medical Systems, Madison, WI, USA) using software version 6.10.029. From this, we derived the total-body areal bone mineral density (aBMD), fat mass (kg), and lean mass (kg). In addition, we were able to obtain the spine region aBMD from the total-body scans. We have previously shown that such scans are equivalent to dedicated lumbar spine DXA measurements in women, with *R* = 0.92 and an error in predicting lumbar spine BMD of 6.5%.(11)

### Central QCT

As described previously,(12,13) single-energy CT scans were made at the lumbar spine and proximal femur with a multidetector Light Speed QX-I scanner (GE Medical Systems, Wakesha, WI, USA). Calibration standards scanned with the patient were used to convert CT numbers directly to equivalent volumetric BMD (vBMD) in milligrams per cubic centimeter (mg/cm^3^).(14) To study age- and sex-specific structural changes in bone mineral distribution and structure, we developed software for the analysis of bone structure, geometry and volumetric density from the CT images, specific details of which have been described previously.(13) To validate our image-processing algorithm, we made 10 scans of the European Spine Phantom, which is composed of hydroxyapatite.(15) The correlation between bone density results determined by our algorithm and that of the spine phantom was *r* = 0.998; using scans of L2 from the phantom over 10 days, vBMD was estimated to have a coefficient of variation (CV) of 0.7%.

### HRpQCT

Details regarding the HRpQCT imaging used in this cohort have been reported previously(16) and are summarized briefly here. Owing to the lack of availability of this new instrument initially, the HRpQCT measurements were done approximately 2 years after the other measurements and in 243 (83%) of the 275 women used in these analyses (80 premenopausal women, 109 postmenopausal women not on HT, and 54 postmenopausal women on HT). The nondominant wrist (or in the case of a prior wrist fracture, the nonfractured wrist) was scanned using an HRpQCT device (a prototype of the XtremeCT, Scanco Medical AG, Bassersdorf, Switzerland). The in vivo measurement protocol included the acquisition of a 3D stack of 116 high-resolution QCT slices at the distal end of the radius using an effective energy of 40 keV, slice thickness of 89 µm, field of view of 90 mm, image matrix of 1024 × 1024 pixels, and pixel size of 89 µm.

The processing and analysis of the images also have been described extensively and validated.(17–20) Briefly, bone volume/total volume (BV/TV) is first derived from the trabecular vBMD. Recognizing that individual trabeculae will not be resolved at their correct thickness owing to partial-volume effects, a thickness-independent structure extraction was employed to assess trabecular microarchitecture. To this end, the 3D ridges (the center points of the trabeculae) were detected in the gray-level images as described in detail in Laib and colleagues.(18) Trabecular number (TbN, 1/mm) then was taken as the inverse of the mean spacing of the ridges.(19) Combining TbN and BV/TV, trabecular thickness (TbTh, mm) then was derived as BV/TV ÷ TbN, and trabecular separation (TbSp, mm) was derived as (1 – BV/TV) ÷ TbN, as is done in standard histomorphometry.(21) The validity of this approach has been rigorously tested by comparing the HRpQCT methodology with 28-µm-resolution micro-CT(20) with very high correlation (correlation coefficients of 0.96–0.99) between the micro-CT and HRpQCT measurements. The key point in this analysis is that the resolution has to be sufficient to adequately resolve the distance between the trabecular ridges (1/TbN, or ∼300 to 500 µm) and not necessarily to resolve individual trabeculae (∼100 µm or less).

### Statistical analysis

Data are summarized as means and SDs. The two-sample *t* test was used for comparisons between groups in Table 1. Unadjusted and age-adjusted Pearson correlation coefficients were used to assess the relationship between serum serotonin levels and the various anthropometric, serum, and skeletal parameters in Table 2. Multivariable models were constructed using age, BMI, and serotonin levels to assess the relative importance of these variables in predicting skeletal parameters. A *P* value of less than .05 was considered significant.

**Table 1:** Anthropometric, Serum, and Bone Density/Structural Parameters in the Study Subjects

			Postmenopausal	
			
	All women	Premenopausal	Not on HRT	On HRT
N	275	90	125	60
Age (years)	57.9 ± 17.7	38.3 ± 9.0	69.3 ± 12.2***	63.7 ± 10.6***††
Anthropometric parameters				
Height (m)	162.2 ± 6.5	164.4 ± 5.7	161.4 ± 6.7**	160.8 ± 6.2***
Weight (kg)	73.2 ± 16.3	71.6 ± 16.5	75.9 ± 16.3	70.1 ± 15.3†
BMI (kg/m^2^)	27.8 ± 5.7	26.4 ± 5.7	29.0 ± 5.3**	27.2 ± 6.1†
Lean mass (kg)	35.8 ± 4.8	37.5 ± 4.4	35.2 ± 5.1**	34.4 ± 4.0***
Fat mass (kg)	33.6 ± 12.5	30.6 ± 13.9	36.7 ± 10.8***	31.7 ± 12.6††
Serum parameters				
Serotonin (ng/mL)	84.9 ± 52.3	95.0 ± 54.5	78.7 ± 52.6*	82.6 ± 46.6
Calcium (mg/dL)	9.4 ± 0.4	9.3 ± 0.3	9.6 ± 0.4***	9.4 ± 0.3††
Phosphorus (mg/dL)	3.5 ± 0.4	3.5 ± 0.5	3.6 ± 0.4*	3.5 ± 0.3
25(OH)D (ng/mL)	22.3 ± 10.1	26.1 ± 12.9	20.2 ± 7.9***	20.8 ± 8.1**
PTH (pmol/L)	3.6 ± 1.6	3.0 ± 1.2	4.0 ± 1.7***	3.7 ± 1.7**
Osteocalcin (ng/mL)	19.7 ± 8.7	19.8 ± 7.8	21.3 ± 9.5	16.2 ± 7.1**†††
PINP (µg/L)	37.8 ± 17.6	38.7 ± 16.5	41.4 ± 19.3	29.1 ± 12.0***†††
CTX (ng/mL)	0.49 ± 0.26	0.48 ± 0.26	0.56 ± 0.26*	0.38 ± 0.21**†††
DXA BMD parameters				
Total-body BMD (g/cm^2^)	1.14 ± 0.11	1.19 ± 0.09	1.11 ± 0.12***	1.13 ± 0.10***
Spine BMD (g/cm^2^)	1.11 ± 0.16	1.16 ± 0.15	1.08 ± 0.16***	1.09 ± 0.15**
Central QCT parameters				
Femur neck				
Total vBMD (mg/cm^3^)	334 ± 74	391 ± 58	296 ± 59***	325 ± 67***††
Trabecular vBMD (mg/cm^3^)	214 ± 59	259 ± 46	184 ± 49***	207 ± 51***††
Cortical vBMD (mg/cm^3^)	581 ± 83	633 ± 70	548 ± 75***	567 ± 77***
Vertebrae				
Trabecular vBMD (mg/cm^3^)	154 ± 44	190 ± 27	130 ± 39***	152 ± 36***†††
HRpQCT parameters^a^				
BV/TV	0.126 ± 0.033	0.136 ± 0.027	0.119 ± 0.035**	0.124 ± 0.033*
TbN (1/mm)	2.50 ± 0.26	2.56 ± 0.19	2.49 ± 0.31	2.46 ± 0.23**
TbTh (mm)	0.050 ± 0.010	0.053 ± 0.008	0.047 ± 0.011***	0.050 ± 0.010
TbSp (mm)	0.354 ± 0.054	0.340 ± 0.035	0.362 ± 0.066**	0.360 ± 0.047**

Data are mean ± SD. BMI = body mass index; 25(OH)D = 25-hydroxyvitamin D; PTH = parathyroid hormone; PINP = amino-terminal propeptide of type I collagen; CTX = cross-linked C-telopeptide of type I collagen; DXA = dual-energy X-ray absorptiometry; BMD = bone mineral density; vBMD = volumetric BMD; BV/TV = bone volume/total volume; TbN = trabecular number; TbTh = trabecular thickness; TbSp = trabecular separation.

a As noted in the “Methods,” the HRpQCT data were available in a subset of the women.

* *P* < .05

** *P* < .01

*** *P* < .001 versus premenopausal women

† *P* < .05

†† *P* < .01

††† *P* < .001 versus postmenopausal women not on HT.

**Table 2:** Unadjusted/Age-Adjusted Correlation Coefficients Between Serum Serotonin Levels and Anthropometric, Serum, and Bone Density/Structural Parameters in the Study Subjects

			Postmenopausal	
			
	All women	Premenopausal	Not on HT	On HT
Age (years)	−0.12*/–	0.08/–	−0.04/–	−0.13/–
Anthropometric parameters				
Height (m)	0.02/−0.02	0.07/0.06	−0.05/−0.08	−0.03/−0.06
Weight (kg)	−0.21***/−0.21***	−0.09/−0.10	−0.30***/−0.33***	−0.14/−0.18
BMI (kg/m^2^)	−0.23***/−0.23***	−0.14/−0.15	−0.32***/−0.34***	−0.13/−0.16
Lean mass (kg)	−0.09/−0.13*	0.03/0.03	−0.21*/−0.24**	−0.16/−0.20
Fat mass (kg)	−0.19**/−0.18**	−0.09/−0.10	−0.27**/−0.30***	−0.11/−0.15
Serum parameters				
Calcium (mg/dL)	0.05/0.08	0.31**/0.33**	−0.00/0.00	0.09/0.08
Phosphorus (mg/dL)	0.08/0.10	0.22*/0.23*	0.09/0.10	−0.15/−0.15
25(OH)D (ng/mL)	0.13*/0.10	0.10/0.12	0.08/0.07	0.18/0.18
PTH (pmol/L)	−0.13*/−0.10	−0.08/−0.09	−0.02/−0.02	−0.32*/−0.31*
Osteocalcin (ng/mL)	0.11/0.10	0.09/0.15	0.16/0.15	0.06/0.02
PINP (µg/L)	0.07/0.05	0.16/0.21*	0.05/0.04	−0.04/−0.08
CTX (ng/mL)	0.04/0.03	0.23*/0.29**	−0.03/−0.03	−0.10/−0.12
DXA BMD parameters				
Total-body BMD (g/cm^2^)	−0.10/−0.17**	−0.09/−0.09	−0.23**/−0.29**	−0.01/−0.06
Spine BMD (g/cm^2^)	−0.12*/−0.16**	−0.20/−0.21*	−0.22*/−0.24**	0.06/0.05
Central QCT parameters				
Femur neck				
Total vBMD (mg/cm^3^)	−0.03/−0.17**	−0.16/−0.15	−0.17/−0.24**	−0.04/−0.09
Trabecular vBMD (mg/cm^3^)	−0.07/−0.25***	−0.25*/−0.26*	−0.17/−0.28**	−0.13/−0.20
Cortical vBMD (mg/cm^3^)	0.06/−0.01	−0.01/−0.01	−0.06/−0.08	0.09/0.06
Vertebrae				
Trabecular vBMD (mg/cm^3^)	0.00/−0.12	−0.15/−0.13	−0.16/−0.22*	0.11/0.07
HRpQCT parameters				
BV/TV	−0.11/−0.16*	−0.10/−0.11	−0.29**/−0.29**	0.06/0.04
TbN (1/mm)	−0.12/−0.16*	−0.06/−0.06	−0.26**/−0.28**	0.03/0.01
TbTh (mm)	−0.11/−0.14*	−0.12/−0.12	−0.26**/−0.26**	0.04/0.03
TbSp (mm)	0.12/0.18**	0.08/0.08	0.28**/0.30**	−0.04/−0.03

Significant (*P* < .05) correlations are indicated in bold. BMI = body mass index; 25(OH)D = 25-hydroxyvitamin D; PTH = parathyroid hormone; PINP = amino-terminal propeptide of type I collagen; CTX = cross-linked C-telopeptide of type I collagen; DXA = dual-energy X-ray absorptiometry; BMD = bone mineral density; vBMD = volumetric BMD; BV/TV = bone volume/total volume; TbN = trabecular number; TbTh = trabecular thickness; TbSp = trabecular separation.

* *P* < .05

** *P* < .01

*** *P* < .001.

## Results

Table 1 shows the anthropometric, serum, and bone density/structural parameters in all the study subjects as well as separately in the premenopausal women, postmenopausal women not on HT, and postmenopausal women on HT. Postmenopausal women not on HT, but not those on HT, had a higher BMI than the premenopausal women. This was due to an increase in fat mass, as determined by total-body DXA. Serum serotonin levels were significantly lower in postmenopausal women not on HT than in premenopausal women; postmenopausal women on HT had intermediate levels not different from either of the two other groups. Serum calcium and phosphorus levels were slightly higher in the postmenopausal women not on HT, and serum 25(OH)D was slightly lower in both groups of postmenopausal women than in premenopausal women, whereas serum PTH levels were significantly higher in both groups of postmenopausal women. Bone formation markers (osteocalcin and PINP) tended to be higher, and serum CTX levels were significantly higher in the postmenopausal women not on HT than in the premenopausal women. Osteocalcin, PINP, and CTX levels were significantly lower in postmenopausal women on HT than in either premenopausal women or postmenopausal women not on HT.

Total-body and spine aBMD values by DXA were significantly lower in both groups of postmenopausal women than in premenopausal women. Similar reductions in the central QCT measures at the femur neck and vertebrae, as well as the distal radius HRpQCT parameters (BV/TV, TbN, and TbTh), were present in the postmenopausal as compared with the premenopausal women, with an increase TbSp in the postmenopausal groups.

**Fig. 1:**
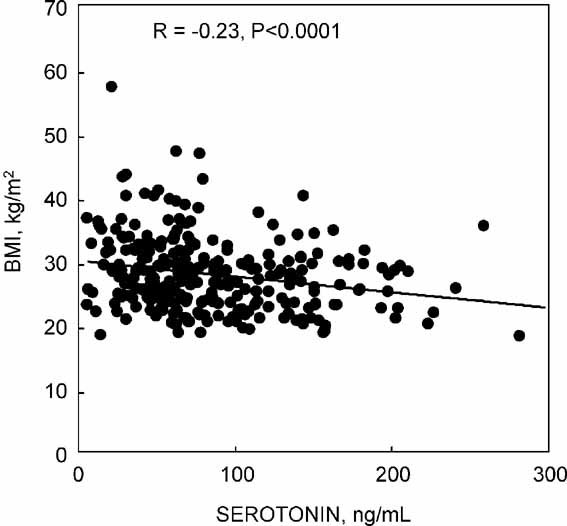
Relation of BMI to serum serotonin levels in all women.

Table 2 shows the unadjusted and age-adjusted correlation coefficients between serum serotonin and the anthropometric, serum, and bone density/structural parameters. Serum serotonin levels were significantly inversely associated with BMI in all women (Fig. 1), with the strongest negative association noted in the postmenopausal women not on HT. This inverse association seemed to be most closely related to fat mass because the correlations between serotonin levels and fat mass were stronger than those between serotonin and lean mass. Since postmenopausal women not on HT had significantly lower serum serotonin levels than premenopausal women but also had higher BMIs (see Table 1), we further tested whether serum serotonin levels were different in the pre- versus postmenopausal women not on HT following adjustment for BMI. In this analysis, differences in BMI were found to account for the differences in serotonin levels between these groups (*P* value following adjustment for BMI = .167).

There were positive associations between serum serotonin and calcium and phosphorus levels in the premenopausal women but not in the other groups (see Table 2). Bone formation and resorption markers tended to be positively associated with serotonin levels, reaching statistical significance for age-adjusted correlations with PINP and unadjusted and age-adjusted correlations with serum CTX levels in premenopausal women.

As also shown in Table 2, there were significant inverse associations between serum serotonin levels and total-body aBMD following adjustment for age in all women. This was driven principally by inverse unadjusted and age-adjusted correlations between serotonin levels and total-body aBMD in the postmenopausal women not on HT. The pattern for DXA spine aBMD was similar, except that at this site the correlations also were statistically significant in premenopausal women following adjustment for age.

Femur neck total vBMD also was inversely associated with serum serotonin levels in all women and in postmenopausal women not on HT following adjustment for age (see Table 2). A similar pattern was seen for femur neck trabecular vBMD, with additional statistically significant inverse associations (unadjusted and age-adjusted) present in premenopausal women. No associations were found between femur neck cortical vBMD and serotonin levels in any of the groups. Vertebral trabecular vBMD was inversely associated with serum serotonin levels in postmenopausal women not on HT following adjustment for age, but not in the other groups.

BV/TV, TbN, and TbTh were inversely and TbSp was positively associated with serum serotonin levels following adjustment for age in all women. As for the DXA and central QCT parameters, these associations were driven principally by the correlations between these microstructural parameters and serotonin levels in postmenopausal women not on HT (see Table 2).

While the data in Table 2 are shown following age adjustment, in additional analyses, we also performed further adjustments for creatinine clearance, serum 25(OH)D, and PTH levels in these subjects. The results remained identical to those shown in Table 2, except that the correlations of serotonin with lean mass in all women and with spine BMD in premenopausal women were no longer significant (data not shown).

**Fig. 2:**
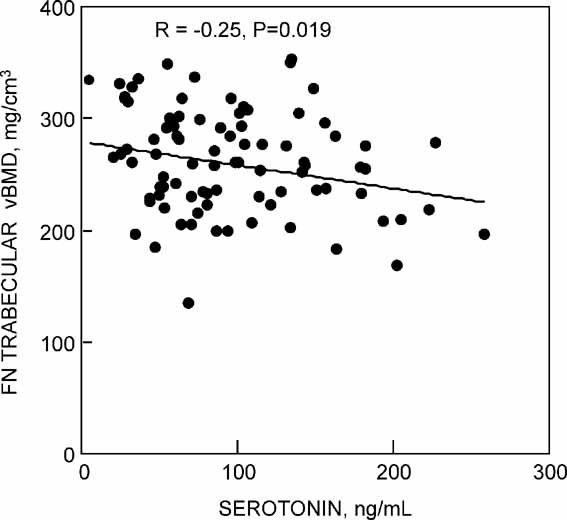
Relation of femur neck trabecular vBMD to serum serotonin levels in premenopausal women.

Since the inverse associations between serotonin and BMI could represent an additional confounder, we constructed multivariable models with bone density and structural parameters as the dependent variables and allowed age, BMI, and serum serotonin levels to compete in these models. Table 3 shows the results of these models for specific skeletal parameters and groups of women where serotonin levels remained as significant predictors. Thus, for femur neck total and trabecular vBMD in all women and trabecular vBMD in premenopausal women, serum serotonin levels remained as significant negative predictors even after age and BMI had entered the models. Since femur neck trabecular vBMD in premenopausal women was significantly inversely associated with serotonin both in the unadjusted (see Table 2) and multivariable models (see Table 3), Figure 2 shows the plot of this relationship. Serum serotonin also remained as a significant negative predictor in models of TbTh in all women, as well as TbTh and BV/TV in postmenopausal women not on HT (see Table 3).

**Table 3:** Multivariable Models in Which Age, BMI, and Serum Serotonin Levels Were Used as Predictors

Outcome	Group	Predictors	β ± SEM	P value	Model R^2^
Femur neck					
Total vBMD (mg/cm^3^)	All women	Age	−2.82 ± 0.20	<.001	0.43
		BMI	1.44 ± 0.65	.028	
		Serotonin	−0.16 ± 0.07	.024	
Trabecular vBMD (mg/cm^3^)	All women	Age	−2.51 ± 0.14	<.001	0.56
		BMI	1.97 ± 0.46	<.001	
		Serotonin	−0.17 ± 0.05	<.001	
Trabecular vBMD (mg/cm^3^)	Premenopausal women	Age	−2.88 ± 0.45	<.001	0.36
		BMI	1.74 ± 0.73	.020	
		Serotonin	−0.16 ± 0.07	.029	
HRpQCT parameters^a^					
TbTh (mm)	All women	Age	−0.14 ± 0.04	<.001	0.05
		Serotonin	−0.03 ± 0.01	.028	
TbTh (mm)	Postmenopausal women not on HT	Serotonin	−0.06 ± 0.02	.007	0.06
BV/TV	Postmenopausal women not on HT	BMI	2.04 ± 0.60	.001	0.16
		serotonin	−0.14 ± 0.06	.038	

*Note:* Shown are the specific skeletal parameters and groups of women where serum serotonin levels were independent predictors in the models. vBMD = volumetric bone mineral density; TbTh = trabecular thickness; BV/TV = bone volume/total volume.

a β and SEM are ×1000.

## Discussion

In this study, we demonstrate significant inverse associations between serum serotonin levels and a number of measures of bone density/structure assessed using a combination of DXA, central QCT, and HRpQCT. For femur neck total and trabecular vBMD and TbTh in all women, for femur neck trabecular vBMD in premenopausal women, and for BV/TV and TbTh at the radius in postmenopausal women not on HT, these associations remained significant in multivariable models that included age and BMI, consistent with an independent effect of circulating serotonin levels on bone mass/structure at these sites. While the associations we found were relatively weak, they were consistent in terms of the direction of the associations in the different groups of women and were statistically significant. Thus our data do support recent findings in rodent models(1,6,7) implicating a physiologic role for serotonin in regulating bone mass. We should note, however, that since we did not correct for possible multiple comparisons, further studies in additional cohorts are needed to validate our findings.

Circulating serotonin is derived principally from the enterochromaffin cells found in gastrointestinal tract crypts and is rapidly taken up by platelets.(22) The serotonin stored in platelets is released during the collection of serum; thus serum serotonin levels are approximately 100-fold higher than platelet-poor plasma levels.(23) It is at present unclear whether serum or platelet-poor plasma serotonin levels are the best “index” of gut serotonin production. In addition, there are different methods available to determine serotonin levels, such as high-pressure liquid chromatography (HPLC)/fluorometry, HPLC/electrochemical, or liquid chromatography-mass spectrometry (LC/MS) techniques. The HPLC assays are considered the gold standard; however, Yadav and colleagues,(1) using the same serotonin assay as used in this study, found that the three OPPG patients tested had approximately fivefold higher serum serotonin levels than the mean of their control subjects; in the same study, two patients with high bone mass owing to activating *LRP5* mutations had an approximately 50% reduction in platelet-poor plasma serotonin levels. Previous studies have found increased serum and platelet-poor plasma serotonin levels in patients with active rheumatoid arthritis compared with control subjects,(23) and immunoassays for serotonin have been used previously in a number of studies in humans.(24,25) Since we only had serum and not platelet-poor plasma in our study subjects, we could not compare serum versus platelet-poor plasma serotonin levels for associations with the bone density/structural parameters. In addition, serum (or platelet-poor plasma) serotonin levels are altered by diet, particularly tryptophan intake,(1) and while all our samples were collected fasting at about 8 a.m., we did not control for dietary factors that might have altered circulating serotonin levels. Thus it is possible that the associations we noted may have been stronger had we used platelet-poor plasma instead of serum for our measurements and been able to control the diet of the study subjects prior to the blood draw.

The study by Yadav and colleagues(1) demonstrated that gut-derived serotonin principally regulated bone formation in vivo and osteoblast proliferation in vitro, with no clear effect on bone resorption parameters in vivo. These findings are in contrast with earlier studies demonstrating that serotonin can enhance osteoclast differentiation in vitro.(26) Interestingly, in our human study we found positive associations between serum serotonin and PINP levels (following age adjustment) as well as between serum serotonin and CTX levels (unadjusted and age-adjusted) in premenopausal women. These findings suggest that, in humans, increased circulating serotonin levels may be associated with increased bone turnover. Thus the effects of serotonin on bone metabolism in humans may be more complex than suggested by the study by Yadav and colleagues,(1) and direct interventional studies (e.g., using serotonin infusions) are likely needed to better define the effects of serotonin in regulating bone formation and resorption in humans.

We did find that serum serotonin levels were inversely associated with BMI and that this association was driven principally by fat mass. The inverse relation between serotonin and body mass has been described previously,(27–31) and it has been suggested that serotonin plays an important role in the regulation of appetite and food satiety, resulting in reduced caloric intake. Furthermore, serotonergic drugs, such as fluoxetine (an SSRI), have been shown to result in significantly greater weight loss than placebo treatment.(32–35) In contrast, blocking serotonin synthesis resulted not only in a prevention of serotonin-induced hypophagia but also an increase in food intake.(36)

We also found that serum serotonin levels were lower in postmenopausal women not on HT than in premenopausal women. However, this does not appear to be an effect of menopause per se because differences in serotonin levels between pre- and postmenopausal women not on HT were no longer significant following adjustment for differences in BMI. We also should note that while we found significant associations between serum serotonin levels and bone mass/structural parameters in postmenopausal women not on HT, these associations were not present in the postmenopausal women on HT. This could be due to biologic effects of HT in modulating the relationship between serotonin and bone or to the smaller sample size of postmenopausal women on HT (*n* = 60) as compared with those not on HT (*n* = 125), and further studies are needed to test whether estrogen may modulate the skeletal effects of serotonin on bone.

In summary, our study does provide support for a possible physiologic role for circulating serotonin levels in regulating bone density/structure in women. However, while statistically significant, the associations we found were relatively weak. This may be due, at least in part, to the fact that we used serum (rather than platelet-poor plasma) for our measurements and did not control the dietary intake of our study subjects. In addition, our data indicate that higher serotonin levels may be associated with increased bone turnover rather than simply reduced bone formation. These caveats notwithstanding, our findings should provide an impetus for additional studies aimed at unraveling the potential role of serotonin in regulating bone metabolism in humans.
